# A Domesticated *PiggyBac* Transposase Interacts with Heterochromatin and Catalyzes Reproducible DNA Elimination in *Tetrahymena*

**DOI:** 10.1371/journal.pgen.1004032

**Published:** 2013-12-12

**Authors:** Alexander Vogt, Kazufumi Mochizuki

**Affiliations:** Institute of Molecular Biotechnology of the Austrian Academy of Sciences (IMBA) Vienna, Austria; University of Utah School of Medicine, United States of America

## Abstract

The somatic genome of the ciliated protist *Tetrahymena* undergoes DNA elimination of defined sequences called internal eliminated sequences (IESs), which account for ∼30% of the germline genome. During DNA elimination, IES regions are heterochromatinized and assembled into heterochromatin bodies in the developing somatic nucleus. The domesticated *piggyBac* transposase Tpb2p is essential for the formation of heterochromatin bodies and DNA elimination. In this study, we demonstrate that the activities of Tpb2p involved in forming heterochromatin bodies and executing DNA elimination are genetically separable. The cysteine-rich domain of Tpb2p, which interacts with the heterochromatin-specific histone modifications, is necessary for both heterochromatin body formation and DNA elimination, whereas the endonuclease activity of Tpb2p is only necessary for DNA elimination. Furthermore, we demonstrate that the endonuclease activity of Tpb2p in vitro and the endonuclease activity that executes DNA elimination in vivo have similar substrate sequence preferences. These results strongly indicate that Tpb2p is the endonuclease that directly catalyzes the excision of IESs and that the boundaries of IESs are at least partially determined by the combination of Tpb2p-heterochromatin interaction and relaxed sequence preference of the endonuclease activity of Tpb2p.

## Author Summary

Transposons are not just threats to genome integrity but also potentially contribute to the evolution of the host. Therefore, host organisms must evolve by balancing the harmfulness and usefulness of transposons. An evolutional product likely created by such a balance is the programmed DNA elimination in the ciliated protist *Tetrahymena*, in which the transposon-related sequences are eliminated by the domesticated *piggyBac* transposase Tpb2p. In this study, we demonstrate that the cysteine-rich domain of Tpb2p interacts with the heterochromatin-specific histone modifications and is necessary for DNA elimination. Furthermore, we demonstrate that the endonuclease activity of Tpb2p in vitro and the endonuclease activity that executes DNA elimination in vivo have similar substrate sequence preferences. These results strongly indicate that Tpb2p is the endonuclease that directly catalyzes the excision of IESs and that the boundaries of IESs are at least partially determined by the combination of Tpb2p-heterochromatin interaction and relaxed sequence preference of the endonuclease activity of Tpb2p. These findings provide a molecular basis of the DNA elimination mechanism as well as of the evolution of a domesticated transposase-mediated genome defense against transposons.

## Introduction

Transposons represent harmful genetic elements because they potentially rearrange their host's genome, and their integration into important coding or regulatory regions can have deleterious effects. Transposons are therefore considered “junk” DNAs [1], and hosts have evolved genome defense mechanisms to counteract these selfish elements [2]. However, transposons may not be just junk because they potentially contribute to the evolution of the host by genome rearrangements, alternating gene expression networks, or providing new genes from transposons to the host [3]. Therefore, host organisms must evolve by balancing the harmfulness and usefulness of transposons. An evolutional product likely created by such a balance is the programmed DNA elimination in the ciliated protist *Tetrahymena*, in which the transposon-related sequences are eliminated by a domesticated *piggyBac* transposase [4].

**Figure 1:**
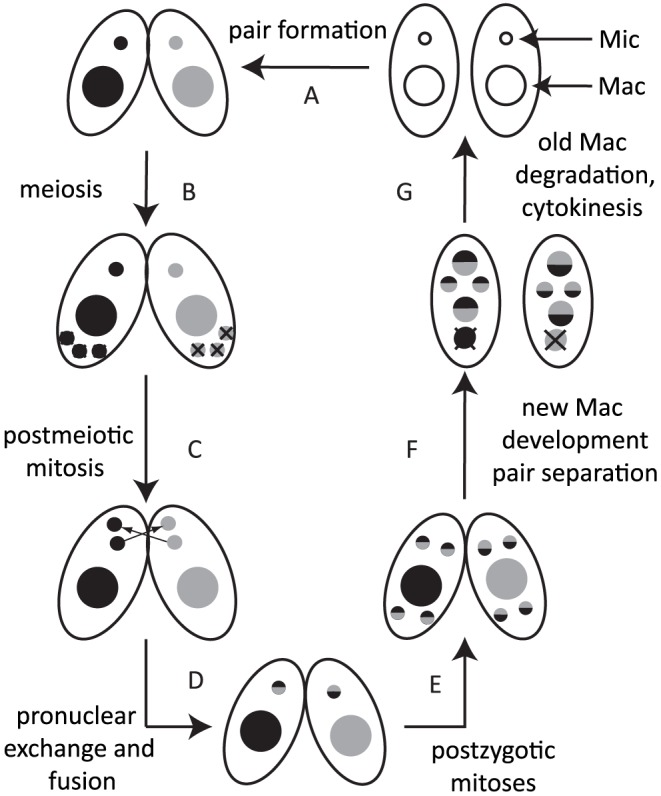
Conjugation of *Tetrahymena*. Under starvation conditions, two *Tetrahymena* cells form a pair (A) and initiate sexual reproduction (conjugation). The Mic undergoes meiosis, and three of the four meiotic products are degraded (B), whereas the remaining product divides mitotically to form two pronuclei in each cell (C). One of the two pronuclei is exchanged with the mating partner, and fusion leads to formation of the zygotic nucleus (D). The zygotic nucleus divides mitotically twice (E); two nuclei become Mics, and the other two differentiate into Macs (F). The parental Mac is degraded. After cell division, the nuclei are distributed to the daughter cells (G).

Most ciliates display nuclear dimorphism [5]. The germline micronucleus (Mic) is transcriptionally inert during vegetative growth, whereas the somatic macronucleus (Mac) provides the cell with most if not all RNA. When nutrients are scarce, *Tetrahymena* undergoes sexual reproduction (conjugation), in which two mating partners form a pair (Fig. 1A) and their Mics undergo meiosis (Fig. 1B). Three of the meiotic products are degraded, and the remaining product divides mitotically (Fig. 1C). One of the products is exchanged with the mating partner and afterwards, the two pronuclei fuse to form the zygote (Fig. 1D). The zygotic nucleus divides twice mitotically (Fig. 1E); of the four mitotic products, two become the new Mics, and the other two develop to become the new Macs (Fig. 1F). The parental Mac is degraded at the end of this process, and the progeny resume vegetative growth when nutrients are supplied (Fig. 1G).

Two major types of programmed genome rearrangements occur in the developing new Mac of *Tetrahymena*. The first type is chromosome breakage, which leads to the fragmentation of germline chromosomes. The chromosome breakage occurs at conserved 15-nt sequences called chromosome breakage sequences (CBSs). It has been estimated that there are ∼250 CBSs in the Mic genome [6], [7]. The sites of chromosome breakages are healed by de novo telomere formation [8]. The second type of genome rearrangement in *Tetrahymena* is DNA elimination of internal eliminated sequences (IESs), followed by ligation of their flanking sequences [9] by the non-homologous end joining (NHEJ) pathway [10]. The indispensability of the NHEJ pathway for DNA elimination was also demonstrated for another ciliate, *Paramecium* [11]. It has been estimated that there are over 8,000 different IESs, which represent ∼30% of the Mic genome [12], [13]. Because many IESs contain transposon-related sequences, it is assumed that DNA elimination is a process that removes potentially harmful transposons from the transcriptionally active somatic genome [14]. Moreover, because some IESs are in regulatory regions and exons of genes, DNA elimination is necessary to create the streamlined functional somatic genome [15]. Despite the fact that different IESs do not share any detectable common sequences within themselves and in their flanking regions, invariable sets of IESs are eliminated from the Mac, and the majority of their boundaries occur within a few to several base pairs.

The identities of IESs are most likely determined epigenetically by an RNAi-related mechanism [16], [17]. While the Mic is transcriptionally inert during the vegetative growth, non-coding RNA transcription occurs in the Mic during the early stages of conjugation. The ∼28–29-nt siRNAs produced from the non-coding RNAs are selected for IES specificity by selective degradation of siRNAs complementary to the parental Mac genome [12]. The selected IES-specific siRNAs eventually induce the establishment of heterochromatin specifically on IESs in the developing new Mac. This heterochromatin comprises tri-methylated histone H3 at lysine 9 and lysine 27 (H3K9me3, H3K27me3) [18], [19] and the chromodomain protein Pdd1p [20], which binds to the histone H3 modifications. Although both H3K9me3 and H3K27me3 have been shown to play an important role in DNA elimination [18], [19], the functional distinction between H3K9me3 and H3K27me3 in the DNA elimination process, if any, is not clear. The heterochromatinized IESs are assembled into heterochromatin bodies located at the nuclear periphery [21], and each IES is eventually excised as one linear or circular piece of DNA [22]–[24]. Artificially tethering Pdd1p to DNA is sufficient to induce DNA elimination [25], indicating that heterochromatin but not the RNAi-related mechanism is the immediate signal inducing DNA elimination.

Previous studies have shown that, in some IES elements, the deletion boundaries are determined by flanking cis-acting sequences located 40–50 bp away, and the precise nature of the deletion is dependent on the sequences at the boundaries [26]–[28]. However, because no sequence homology has been observed across different elements, it is unclear how the boundaries of IESs are determined. In addition it is not known whether and how heterochromatin is involved in the boundary determination. We previously reported that the domesticated *piggyBac* transposase-like protein Tpb2p (Tetrahymena *piggyBac*-like transposase 2) localizes to the heterochromatin bodies and is essential for DNA elimination [4]. Furthermore, we reported that Tpb2p has the ability to produce DNA double-strand breaks at a boundary sequence of an IES in vitro [4]. Therefore, we hypothesized that Tpb2p is recruited to the IESs by directly interacting with a heterochromatin component and then inducing a DNA double-strand break at its preferential DNA sequence near the heterochromatin to execute DNA elimination. To validate this hypothesis, we analyze the roles of the individual domains of Tpb2p genetically and biochemically to understand 1) how Tpb2p interacts with heterochromatin; 2) whether the Tpb2p-heterochromatin interaction is necessary for DNA elimination; 3) what is the sequence preference of the endonuclease activity of Tpb2p; and 4) whether the sequence preference contributes to the choice of boundary sequence of IESs. Based on the results, we discuss how Tpb2p is involved in reproducible DNA elimination and how a domesticated transposase has evolved to catalyze the DNA elimination of transposons.

## Results

### Conditional knockout of *TPB2* phenocopies *TPB2* loss via RNAi knockdown

Recombinantly expressed Tpb2p has been demonstrated to have the ability to produce DNA double-strand breaks in vitro [4]. However, it is unclear whether the endonuclease activity of Tpb2p is necessary for DNA elimination in vivo because Tpb2p is also necessary for the formation of the heterochromatin bodies, which is believed to be a prerequisite of DNA elimination. To test whether the endonuclease activity of Tpb2p is needed for DNA elimination in vivo, we attempted to express a catalytically inactive Tpb2p mutant in a *TPB2*-null background. RNAi knockdown, which has been previously used to study the function of *TPB2* [4], only partially down-regulates *TPB2* expression and is difficult to use for genetic rescue experiments. Therefore, we attempted to obtain knockout (KO) strains of *TPB2* but without success. This lack of success might be because *TPB2* is a haplo-insufficient gene, and heterozygous *TPB2* KO strains are not viable. This is consistent with the fact that RNAi knockdown of *TPB2* caused nearly complete lethality of sexual progeny [4]. To overcome this problem, we created *TPB2* conditional knockout (cKO) strains.

**Figure 2:**
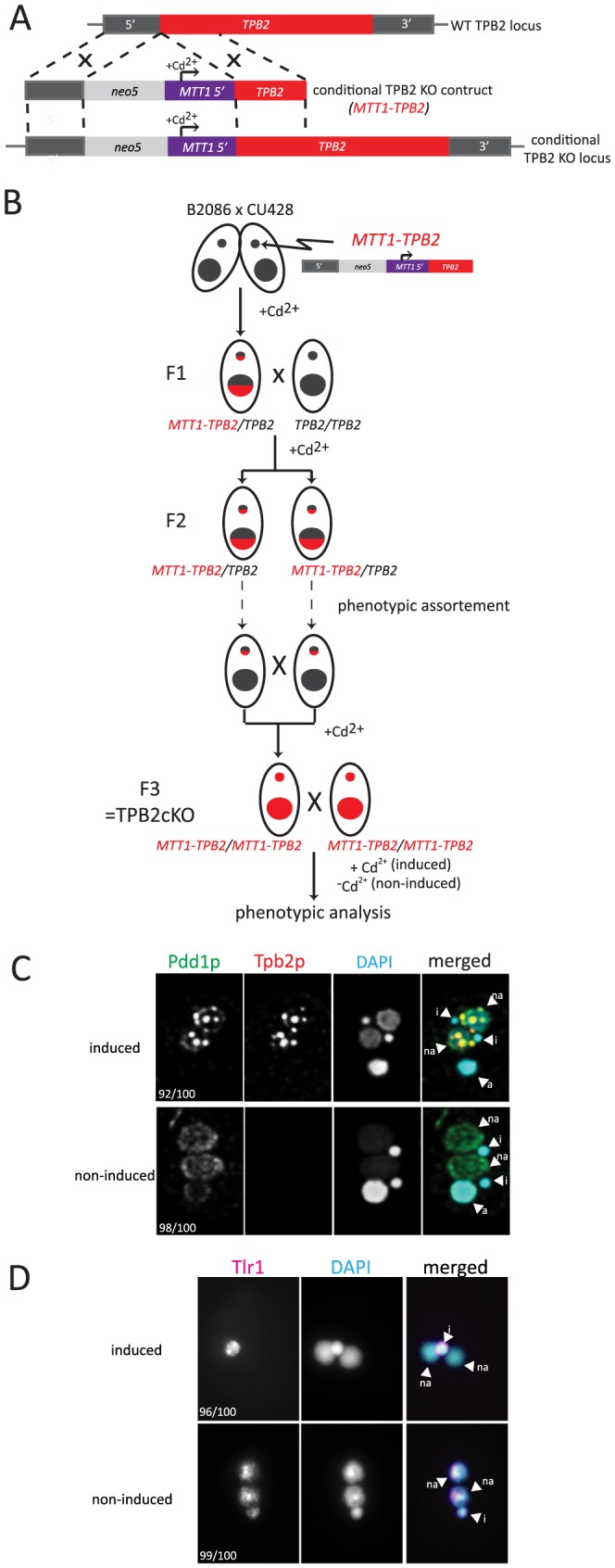
Construction and analyses of *TPB2* conditional KO (cKO) cells. (A) The *TPB2* conditional KO construct (*MTT1-TPB2*) was introduced into the endogenous *TPB2* locus (WT *TPB2* locus) by homologous recombination to create the *TPB2* conditional KO locus, in which *TPB2* expression is under the control of the cadmium-inducible *MTT1* promoter. (B) Germline transformation with the *MTT1-TPB2* construct resulted in one heterozygous *TPB2* cKO clone (F1), which was then crossed with WT to obtain the heterozygous *TPB2* cKO clones with different mating types (F2). The heterozygous *TPB2* cKO clones were phenotypically assorted (dotted arrows) until they lost Mac copies of *MTT1-TPB2* and afterwards crossed with each other to obtain homozygous *TPB2* cKO strains (F3). (C) Mating *TPB2* cKO cells were incubated with (induced) or without (non-induced) cadmium, and exconjugants (progeny) were fixed at 14 hr post-mixing for immunofluorescence staining. The cells were triple stained with a guinea pig anti-Pdd1p antibody (green), which marks heterochromatin, a rabbit anti-Tpb2p antibody (red) and DAPI (blue), which stains DNA. The number of cells displaying the phenotype represented by the pictures in each culture condition are shown. (D) Mating *TPB2* cKO cells were incubated with (induced) or without (non-induced) cadmium, and exconjugants (progeny) were fixed at 36 hr post-mixing for DNA FISH against the Tlr1 IES to assess DNA elimination. DNA was counterstained with DAPI. The number of cells displaying the phenotype represented by the pictures in each culture condition are shown. i = micronucleus; na = new macronucleus.

To make *TPB2* expression conditional, we produced a *TPB2* cKO construct in which the endogenous *TPB2* promoter was replaced with the cadmium-inducible *MTT1* promoter (Fig. 2A). The *TPB2* cKO construct was first introduced into the *TPB2* locus in the Mic by homologous recombination to produce heterozygous *TPB2* cKO strains, and then, two heterozygous *TPB2* cKO strains were mated to obtain homozygous *TPB2* cKO strains (hereafter referred to as *TPB2* cKO strains) (Fig. 2B). In these genetic crosses, *TPB2* expression was continuously induced during conjugation to obtain viable progeny.

**Figure 3:**
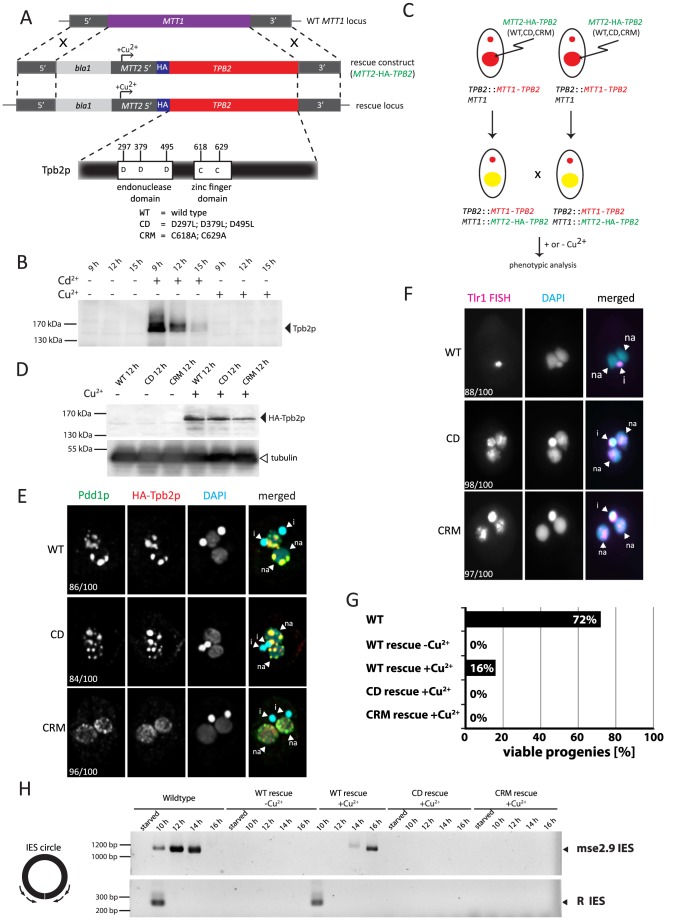
Functional analyses of the endonuclease and cysteine-rich domains of *TPB2* in vivo. (A) The *TPB2* rescue construct (*MTT2-TPB2*) was introduced into the endogenous *MTT1* locus (WT *MTT1* locus) by homologous recombination to create the *TPB2* rescue locus, in which *TPB2* expression is under the control of the copper-inducible *MTT2* promoter. Three different *TPB2* rescue constructs encoding for wild-type Tpb2p (WT), catalytic-dead Tpb2p (CD) and a cysteine-rich mutant Tpb2p (CRM) were used. (B) *TPB2* cKO strains were mated and either left uninduced or cadmium or copper were added during conjugation. Tpb2p expression from the *MTT1* promoter at the TPB2 cKO locus at 9, 12 and 15 h post-mixing was detected by western blot using an anti-Tpb2p antiserum. (C) Scheme of production of the *TPB2* rescue strains. A *TPB2* rescue construct was introduced into the *MTT1* locus of the Mac of *TPB2* cKO cells. (D) *TPB2* rescue strains expressing WT, CD or CRM *TPB2* were mated, and Tpb2p expression from the MTT2 promoter at the rescue locus was compared between in the absence (−) or presence (+) of CuSO_4_ by western blot (top). As a loading control, expression of alpha-tubulin was analyzed (bottom) (E) Mating *TPB2*-rescued cells with wild-type (WT), catalytic-dead (CD) or cysteine-rich mutant (CRM) construct were incubated with copper, and exconjugants (progeny) were fixed at 14 hr post-mixing for immunofluorescence staining. The cells were triple stained with a guinea pig anti-Pdd1p antibody (green), which marks heterochromatin, a rabbit anti-Tpb2p antibody (red), and DAPI (blue), which stains DNA. The numbers of cells displaying the phenotype represented by the pictures in each culture condition are shown. (F) Mating *TPB2* rescued cells were incubated with CuSO_4_, and exconjugants (progeny) were fixed at 36 hr post-mixing for DNA FISH against the Tlr1 IES to assess DNA elimination. DNA was counterstained with DAPI. The number of cells displaying the phenotype represented by the pictures in each culture condition are shown. i = micronucleus; na = new macronucleus. (G) Viability of sexual progeny of the wild-type (WT)or the *TPB2* rescue strains without (−Cu^2+^) or with (+Cu^2+^) induction of the wild-type (WT rescue), catalytic-dead (CD rescue) or cysteine-rich mutant (CRM rescue) *TPB2* were analyzed. 192 single mating pairs were placed into drops of medium and incubated for ∼60 h at 30°C. Completion of conjugation was confirmed by testing for negative expression of the marker specific for the parental Macs. (H) Genomic DNA was extracted from starved or mating (10, 12, 14 and 16 h post-mixing) cells of the wild-type or the *TPB2* rescue strains without (−Cu^2+^) or with (+Cu^2+^) induction of the wild-type (WT), catalytic-dead (CD) or cysteine-rich mutant (CRM) rescue constructs, and circularized mse2.9- and R-IES elements were detected by nested PCR as shown on the left.

It is known that the *MTT1* promoter is leaky in the standard culture conditions [29]. Therefore, we used a metal-depleted medium (see Materials and Methods for details) to minimize the basal level activity of the *MTT1* promoter. Western blot analysis and immunofluorescent staining using an anti-Tpb2p antibody demonstrated that in our culture conditions, Tpb2p was undetectable in the absence of cadmium during conjugation (see Fig. 3B −Cd^2+^ lanes) of the *TPB2* cKO strains. In contrast, when cadmium was added, Tpb2p expression was clearly detected (see Fig. 3B +Cd^2+^ lanes). Therefore, Tpb2p expression can be conditionally knocked out in the *TPB2* cKO strains.

It has been reported that the formation of heterochromatin bodies and DNA elimination can be inhibited by the RNAi knockdown of *TPB2* [4]. To determine if the cKO of *TPB2* phenocopies the RNAi knockdown of *TPB2*, the formation of heterochromatin bodies in the *TPB2* cKO cells was observed by immunofluorescent staining of the heterochromatin component Pdd1p. When *TPB2* expression was induced in the presence of cadmium, the *TPB2* cKO strains formed Pdd1p-containing heterochromatin bodies in the new Macs (Fig. 2C, “induced”). In contrast, in the absence of *TPB2* induction, Pdd1p-stained heterochromatin did not form large bodies but remained as dispersed small foci in the new Mac (Fig. 2C, “non-induced”).

Next, DNA elimination in *TPB2* cKO strains was observed by DNA fluorescence in situ hybridization (FISH) against Tlr1 IESs, which are moderately repeated (∼30 copies) in the Mic genome [30]. DNA elimination in wild-type cells is normally completed by ∼16-hr post-mixing [31]. In the presence of cadmium, Tlr1 IESs were undetectable in the new Macs at 36-hr post-mixing and detected only in the Mic (Fig. 2D, “induced”, na = new Mac, i = Mic), indicating that these IESs were removed completely from the new Macs. In contrast, the Tlr1 IESs remained in the new Mac even at 36-hr post-mixing when *TPB2* expression was not induced (Fig. 2D, “non-induced”). These results indicate that the *TPB2* cKO strains exhibit defects in heterochromatin body formation and DNA elimination in the absence of the induction of *TPB2*, as it was previously reported for the *TPB2* RNAi knockdown strains.

### Establishment of a genetic rescue system to study the function of Tpb2p domains

Next, we attempted to establish a genetic rescue system in which the non-essential *MTT1* locus [29] of the parental Mac in the *TPB2* cKO strains was replaced with a *MTT2* cassette expressing a gene of interest under the control of the copper-inducible *MTT2* promoter [32] (Fig. 3A). Before starting the rescue experiments, we first tested whether the *TPB2* cKO locus could be kept silent in the presence of copper. We incubated the conjugating *TPB2* cKO cells either with cadmium or copper, and Tpb2p expression was analyzed by western blot using an anti-Tpb2p antibody. Although Tpb2p expression was induced in the presence of cadmium, it was undetectable from the cells incubated with copper (Fig. 3B). These results indicate that the expression of *TPB2* in the *TPB2* cKO locus, which is under control of the cadmium-inducible *MTT1* promoter, is not induced by the addition of copper.

Next, the *MTT2* cassette containing the wild-type *TPB2* tagged with HA epitope (Fig. 3A, *MTT2*-HA-*TPB2*) was introduced into the *TPB2* cKO strains (Fig. 3C), and the cells were incubated with or without copper. We analyzed HA-Tpb2p expression by western blot using an anti-HA antibody and observed that HA-Tpb2p was detected only when copper was added to the medium (Fig. 3D, compare WT +/− Cu^2+^). Therefore, the expression of a gene in the *MTT2* cassette is induced in the presence of copper. When the *MTT2* cassette containing the wild-type *TPB2* was introduced into the *TPB2* cKO strains, heterochromatin body formation (Fig. 3E, WT) and DNA elimination (Fig. 3F, WT) were restored in the presence of copper. Also, expression of the wild-type *TPB2* partially restored the progeny viability of the *TPB2* cKO strains (Fig. 3G, WT-rescue +Cu^2+^). These results indicate that the wild-type *TPB2* expressed from the *MTT2* cassette in the parental Mac is sufficient for all essential steps of DNA elimination, although it might not be enough to restore some non-essential steps of DNA elimination. Therefore, the rescue system using the *TPB2* cKO strains and *MTT2* cassette can be used to assay functionalities of Tpb2p mutants.

DNA elimination process was also analyzed by observing circularized excised IESs by PCR (see Fig. 3H left). DNA elimination events in the wild-type cells release IESs in two different forms: the major linear form and the minor circular form [23], [24]. The circular form of two different IESs, mse2.9 and R elements, were detected when the wild-type *TPB2* was expressed in the *TPB2* cKO background (Fig. 3H, WT rescue +Cu^2+^). However, the appearance of the excised mse2.9 IES was delayed in the conditional *TPB2* KO cells expressing the wild-type *TPB2* compare to the wild-type cells (Fig. 3H, see Wild-type and WT rescue +Cu^2+^), possibly because the ectopic expression of *TPB2* from the *MTT2* promoter in the parental Mac could not fully restore the function of endogenous *TPB2*. This may explain why the progeny viability was much lower in the conditional *TPB2* KO cells expressing the wild-type *TPB2* than in the wild-type cells (Fig. 3G).

### The endonuclease activity of Tpb2p is essential for DNA elimination but not for heterochromatin body maturation

Tpb2p has the endonuclease catalytic domain that contains three aspartic acids that form the DDD catalytic triad (Fig. 3A). We previously reported that the replacement of these three aspartic acids with leucines compromises the endonuclease activity of Tpb2p in vitro [4]. To understand the role of the endonuclease activity in vivo, the *MTT2* cassette expressing the *TPB2*-CD mutant, in which the catalytic triad of Tpb2p was replaced with leucines (D297L; D379L; D495L, “CD” in Fig. 3A), was introduced into the *TPB2* cKO strains. The amount of Tpb2p-CD expressed from the *MTT2* cassette after induction with copper was comparable to that of the wild-type Tpb2p from the cassette (Fig. 3D, compare +Cu^2+^ lanes of WT and CD), indicating that the mutations do not significantly affect the stability of Tpb2p. The expression of the *TPB2*-CD mutant did restore heterochromatin body maturation, based on the localization of the heterochromatin component Pdd1p (Fig. 3E, “CD”), but did not support the elimination of Tlr1 IESs from the new Macs, as evaluated by the fact that FISH using the probes complementary to Tlr1 IESs stains the new Mac (Fig. 3F, “CD”). The circular form of two different IESs, mse2.9 and R elements, could not be detected by PCR when *TPB2*-CD expression was induced (Fig. 3H, CD rescue +Cu^2+^), indicating that no detectable IES excision was induced by the *TPB2*-CD expression. Consistent with the fact that DNA elimination is essential for the production of viable sexual progeny, expression of *TPB2*-CD mutant could not restore the progeny viability of the *TPB2* cKO strains (Fig. 3G). Altogether, we conclude that the endonuclease activity of Tpb2p is necessary for DNA elimination but dispensable for the heterochromatin body formation.

### The cysteine-rich domain of Tpb2p is essential for heterochromatin body formation and DNA elimination

The results above clearly indicate that the necessity of Tpb2p for heterochromatin body formation (Fig. 2C) should be attributed to an activity of Tpb2p other than its endonuclease activity. The endonuclease domain of *piggyBac* transposases is followed by a PHD finger-like domain [33], [34] (Supplemental Fig. S1). *Tetrahymena* Tpb2p and its *Paramecium* homolog Pgm [35] also have a cysteine-rich domain downstream of their endonuclease domains (Fig. 3A, Supplemental Fig. S1). Although this cysteine-rich domain displays similarity to the PHD finger domain, it lacks a potential metal-binding residue in one of the two intermingled zinc-fingers (Supplemental Fig. S1). Therefore, it is unclear whether the cysteine-rich domain of Tpb2p has any biological role or if it is only a non-functional remnant of the PHD finger domain of the ancestral *piggyBac* transposase.

To determine if the cysteine-rich domain of Tpb2p has any role in DNA elimination, a *MTT2* cassette containing the *TPB2*-CRM mutant, in which two of the seven potential metal-binding core cysteine/histidine residues of the cysteine-rich domain were replaced with alanines (C618A; C629A, “CRM” in Fig. 3, Supplemental Fig. S1), was introduced into the *TPB2* cKO strains. The amount of Tpb2p-CRM expressed after induction with copper was comparable to that of the wild-type Tpb2p from the cassette (Fig. 3D, compare +Cu^2+^ lanes of WT and CRM), indicating that the mutations did not significantly affect the stability of Tpb2p. We observed that the expression of *TPB2-CRM* did not restore heterochromatin body formation (Fig. 3E, CRM), the elimination of Tlr1 IESs from the new Macs (Fig. 3F, CRM), the formation of circularized excised IESs (Fig. 3H, CRM rescue +Cu^2+^), and the progeny viability (Fig. 3G, CRM rescue +Cu^2+^). Therefore, we concluded that the cysteine-rich domain of Tpb2p is essential for both heterochromatin body formation and the following DNA elimination.

### Heterochromatin-specific histone H3 methylations enhance the interaction between the cysteine-rich domain of Tpb2p and the N-terminal tail of histone H3

The cysteine-rich domain of Tpb2p resembles the PHD-finger domain (Supplemental Fig. S1), and some PHD finger-containing proteins bind to the N-terminal tail of histone H3 with methylated lysine residues [36]. Because histone H3 on the heterochromatinized IESs is specifically tri-methylated at lysine 9 and lysine 27 (H3K9me3/K27me3), we reasoned that Tpb2p might interact with heterochromatin through the interaction between its cysteine-rich domain and H3K9me3/K27me3. This hypothesis is consistent with the fact that although the wild-type Tpb2p and Tpb2p-CD tightly co-localized with the heterochromatin component Pdd1p (Fig. 3E WT, CD), the localization of Tpb2p-CRM did not completely overlap with that of Pdd1p (Fig. 3E, CRM). Therefore, we aimed to investigate if the cysteine-rich domain of Tpb2p interacts with H3K9me/K27me.

We prepared C-terminally biotinylated peptides representing the amino acids 1–19 or 16–35 of *Tetrahymena* histone H3 (Supplemental Table S2). The peptides were either unmodified or tri-methylated at lysine 4, 9 or 27. In addition, unmodified peptides with scrambled amino acid orders were prepared. The peptides were bound to avidin-coated beads and incubated with a recombinant cysteine-rich domain (aa 566–aa 657) of Tpb2p fused to a maltose-binding protein (MBP)-tag (MBP-Tpb2p-CRD), and the proteins co-precipitated with the peptides were analyzed by western blotting using an anti-MBP antibody.

**Figure 4:**
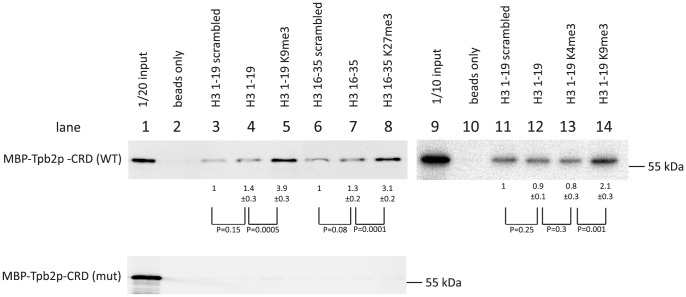
In vitro histone peptide-binding assay. Indicated biotin-tagged histone peptides were immobilized on streptavidin Dynabeads and incubated with the recombinant expressed MBP-tagged cysteine-rich domain (566 aa–657 aa) of Tpb2p (MBP-Tpb2p-CRD (WT)) or peptides having mutations (C618A, C629A) at the putative metal-binding cysteines (MBP-Tpb2p-CRD (mut)). Proteins co-precipitated with the histone peptides were detected by western blot using anti-MBP antiserum. The average intensities of bands from three independent experiments were normalized to the value of “scrambled” peptides. Standard deviations are shown after the average intensity values. P-values from a student's T-test are shown.

We observed that more MBP-Tpb2p-CRD was precipitated with the unmodified peptides than with the beads only (compare lanes 2 and 4, lanes 2 and 7 or lanes 10 and 12 of Fig. 4, top), whereas the amount of MBP-Tpb2p-CRD precipitated with these unmodified peptides was comparable to the amount precipitated with the corresponding unmodified peptides with scrambled amino acid orders (compare lanes 3 and 4, lanes 6 and 7 or lanes 11 and 12 of Fig. 4, top). Therefore, some physical property of the peptides, such as charge, likely mediates the co-precipitation of MBP-Tpb2p-CRD with the unmodified peptides. Importantly, significantly more MBP-Tpb2p-CRD was co-precipitated with the peptides having tri-methylated lysines 9 or 27 than with the corresponding unmodified peptides (compare lanes 4 and 5 or lanes 7 and 8 of Fig. 4, top). Therefore, the presence of tri-methylations at lysine 9 or lysine 27 enhances the interaction between MBP-Tpb2p-CRD and the peptides. In contrast, tri-methylations at lysine 4 did not enhance co-precipitation of MBP-Tpb2p-CRD with the peptide (compare lanes 12 and 13 of Fig. 4). The mutations (C618A, C629A) at the putative metal-binding core of the cysteine-rich domain, which abolish the heterochromatin body formation in vivo (Fig. 3E), inhibited the co-precipitation of MBP-Tpb2p-CRD with any of the peptides in vitro (Fig. 4, bottom). We conclude that Tpb2 can interact with the histone H3 tail through its cysteine-rich domain, and this interaction is significantly enhanced by the presence of tri-methylated lysines 9 or 27.

### The integrity of the left R-IES boundary sequence is important for precise cleavage by Tpb2p in vitro

**Figure 5:**
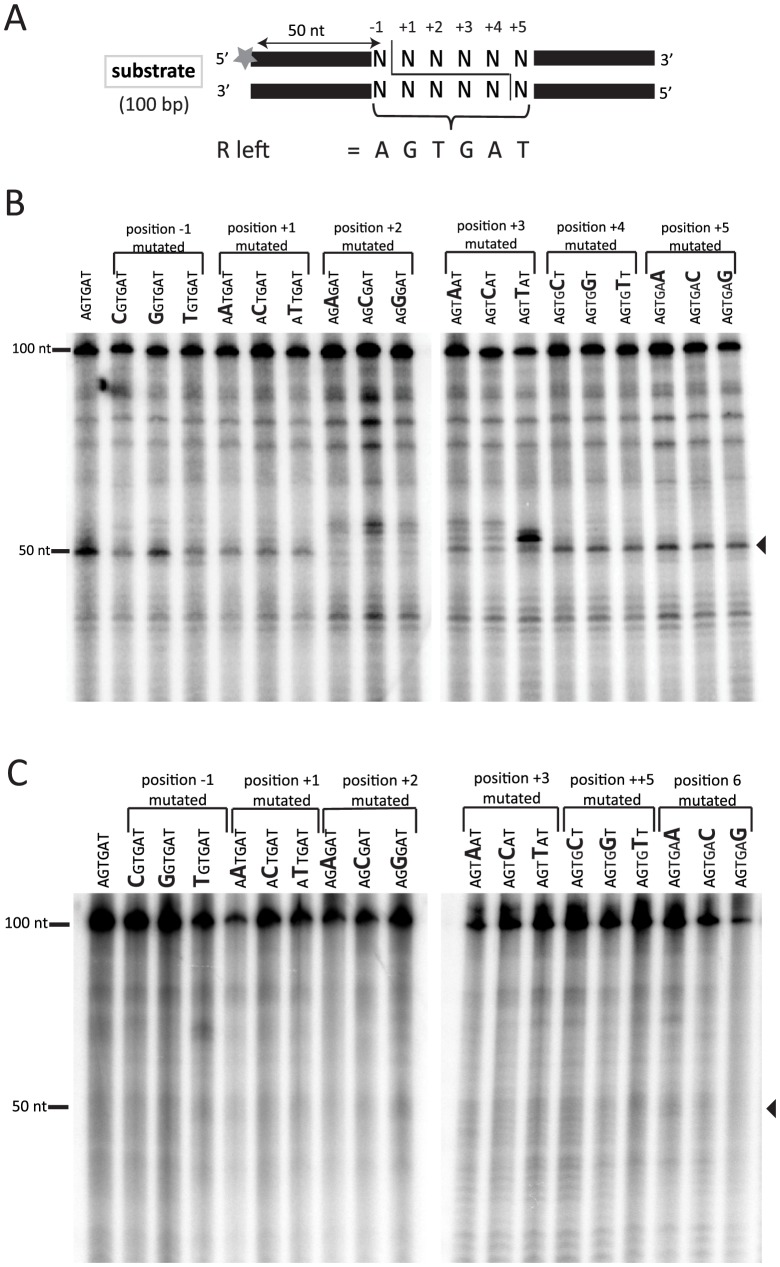
In vitro Tpb2p endonuclease assay. (A) Schematic drawing of the oligo DNA substrates. The substrates were 100 bp long, and 6 bp boundary sequences from the R-IES (5′-AGTGAT-3′) or those having mutated versions of the boundary, in which every position was substituted to every other possible nucleotide, were placed at the 50^th^ to 55^th^ position, which were designated positions −1 to +5. The top strands of the substrates were ^32^P-labeled at their 5′-ends. The expected cleavage site, where a 4-base 5′-overhang DSB is expected to occur during DNA elimination in vivo, is after the 50^th^ nucleotide. (B, C) The substrates were incubated with the wild-type Tpb2p (B) or the catalytic dead Tpb2p (Tpb2p-CD) (C) expressed recombinantly in *E. coli*. The products were separated in a denaturing polyacrylamide gel and visualized by autoradiography. The positions of the 50 and 100 nt markers separated in the same gel are shown on the left.

We have previously shown that recombinant Tpb2p, which is expressed from *E. coli*, produces DNA double-strand breaks (DSB) possessing 4-base 5′ overhangs at the left boundary sequence of *Tetrahymena* R-IES (5′-AGTGAT-3′) in vitro [4] (see also Fig. 5B). However, it is unclear what sequence feature, if any, is recognized by Tpb2p. To better understand what sequence feature of an IES boundary is recognized by Tpb2p, the left R-IES boundary sequence (5′-AGTGAT-3′) was placed in the middle of otherwise artificial sequence, and every position of the boundary was substituted with every other possible nucleotide (Fig. 5B). The radiolabeled oligo DNA duplexes were incubated with recombinant Tpb2p and analyzed as described above. As previously observed, when Tpb2p was incubated with an oligo DNA duplex having the wild-type R-IES boundary, the major 50-nt product, which is produced by the endonucleolytic cleavage between the first A and first G of the boundary (see Fig. 5A), was detected (Fig. 5B, the leftmost lane). Base substitutions at positions −1, +1, +4 and +5 did not significantly affect the choice of cleavage position by Tpb2p in this in vitro assay (Fig. 5B). In contrast, when position +2 was substituted, the major 50-nt product was greatly reduced, and instead, a few nucleotide longer products were detected (Fig. 5B). Similarly, when position +3 was substituted, a few nucleotide longer products were detected, although the 50-nt product was not significantly reduced (Fig. 5B). Importantly, no obvious cleavage products were detected when the substrates were incubated with the catalytically inactive Tpb2p (Fig. 5C), indicating that the observed products were produced by Tpb2p, but not by any contaminated bacterial endonucleases. All together, these results suggest that most nucleotides of the left R-IES boundary sequence are replaceable without disturbing the boundary recognition by Tpb2p, whereas the 5′-TG-3′ sequence at positions +2 and +3 are important for Tpb2p to execute precise cleavage of the left R-IES boundary in vitro.

Because not all boundary sequences of IESs share 5′-TG-3′ at their +2 and +3 positions [22], [37], [38], this dinucleotide sequence cannot be the sole sequence feature recognized by Tpb2p. Tpb2p may recognize multiple different sequence features, including 5′-TG-3′. Alternatively, Tpb2p may recognize some complex combinatorial sequence feature, which is shared in all boundary sequences of IESs and was not completely disrupted by the single-base substitutions in this study, and 5′-TG-3′ may be a part of this combinatorial sequence feature. From this study, we can conclude that the endonuclease activity of Tpb2p has a relaxed but not completely identified sequence preference for its substrates.

### The in vivo effect of base replacement of the left R-IES boundary sequence strongly indicates that Tpb2p is the excisase

Although our genetic analyses indicated that the endonuclease activity of Tpb2p is required for DNA elimination (Fig. 3), none of the results directly demonstrated that Tpb2p is the “excisase,” the enzyme that cuts out IESs in vivo. Above, we observed that the base substitution of +2 or +3 positions of the left R-IES boundary force Tpb2p to cleave a few bases downstream in vitro (Fig. 5B). If the same base substitutions at the left R-IES boundary forced a shift in the position of DSB downward in vivo, it would support the assignation of the excisase function to Tpb2p.

**Figure 6:**
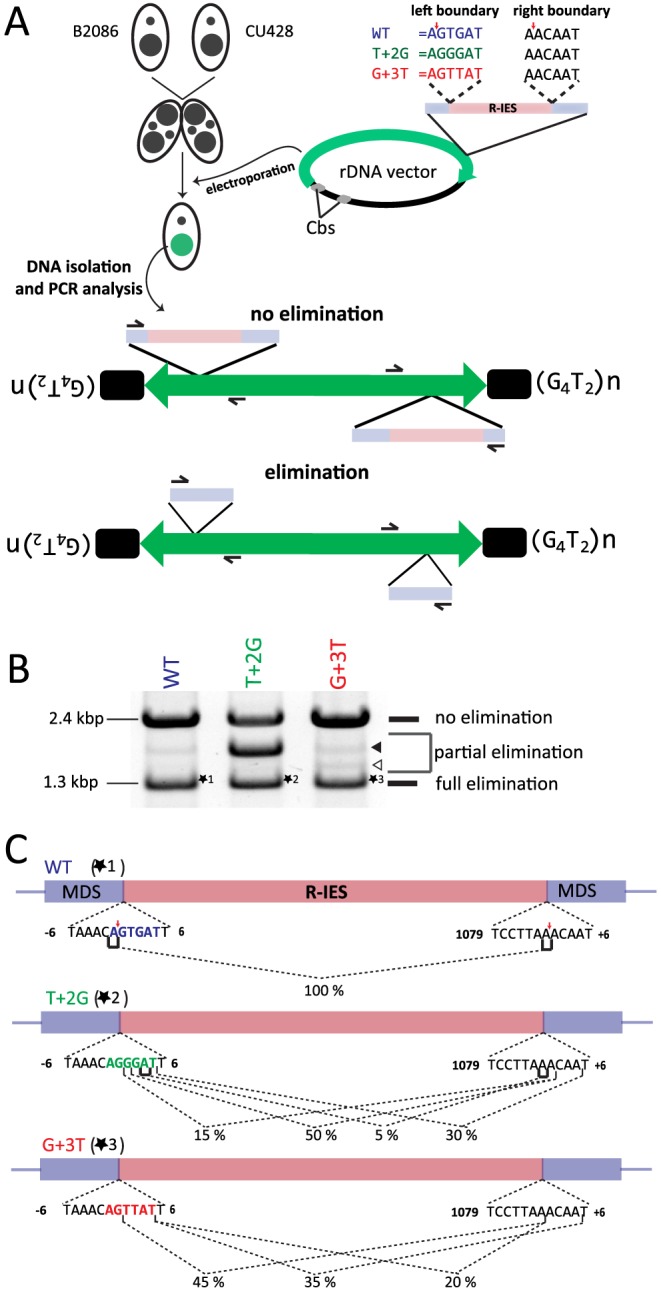
In vivo elimination assay using mutated R-IES boundaries. (A) Two wild-type strains (B2086 and CU428) were mated, and the rDNA vector containing the R-IES and its flanking regions was introduced into the new Mac of their progeny. The left boundary of the R-IES was WT, the position +2 T was mutated to G (T+2G) or the position +3 G was mutated to T (G+3T). The introduced circular rDNA vector was rearranged into an rDNA “minichromosome” in which two copies of rDNA are joined in inverted orientations and telomeres are formed at the ends. The R-IES inserted in the 3′ non-coding region of the rDNA is subjected to DNA elimination similar to the endogenous R-IES [26]. The reported cleavage positions at the endogenous R-IES locus are indicated by red arrows. (B) Twenty-four progeny from each construct were pooled, and their genomic DNA was analyzed by PCR using the primer set shown in (A) to observe the elimination of R-IES from the rDNA. The PCR products were separated by agarose gel electrophoresis. The quickly migrating products (∼1.3 kbp, marked with *1, *2 or *3) correspond with the rDNA locus where the full (or nearly full) length of R-IES was eliminated (full elimination). The most slowly migrating product (∼2.4 kbp) bands correspond with the rDNA locus where no R-IES elimination occurred (no elimination). Some products (open and closed arrowheads) migrating between the two products correspond with the rDNA locus where the R-IES were partially eliminated (partial elimination) (C) The ∼1.3-kbp PCR products, marked with *1, *2 or *3 in (B), were cloned, and sequences of 48 clones from each rDNA construct were analyzed to assign cleavage sites. The lines under the boundary sequences indicate the positions where the cleavage sites were assigned at exact positions. The brackets indicate the positions where the cleavage site could not be mapped at exact positions due to sequence redundancies (direct repeats). The fractions of the cleavage sites among the 48 observed clones are shown. The reported cleavage positions at the endogenous R-IES locus are indicated by red arrows.

A previously established, in vivo IES elimination assay was used to test this possibility. It has been demonstrated that IESs introduced into a non-coding region of the ribosomal DNA (rDNA) are removed precisely as their endogenous counterparts, albeit with lower efficiency, when the rDNA construct is introduced into the developing new Mac [26]. We prepared three different rDNA constructs (Fig. 6A, right top) having the R-IES and its flanking regions with 1) no base substitution (WT construct); 2) T to G substitution at position +2 of the left boundary (T+2G construct); and 3) G to T substitution at position +3 of the left boundary (G+3T construct). These constructs were introduced into the new Mac of conjugating wild-type cells by electroporation (Fig. 6A). Twenty-four sexual progeny possessing the transgenic rDNA were pooled for each construct, and their genomic DNA was analyzed by PCR to observe elimination of the R-IES of the introduced rDNA (Fig. 6A, bottom).

First, the PCR products were analyzed by gel electrophoresis. Two major PCR products were detected from the cells transformed with the WT construct (Fig. 6B, WT). The shorter (1.3 kbp) and longer (2.4 kbp) major products correspond with the R-IES locus on rDNA with or without IES elimination, respectively. A minor PCR product (1.8 kbp), in which a part of R-IES was eliminated (data not shown), was also detected (Fig. 6B, WT, closed arrowhead). Similar PCR products were also detected from cells transformed with the T+2G construct (Fig. 6B, T+2G) and the G+3T construct (Fig. 6B, G+3T). In the cells transformed with the T+2G construct, 1.8-kbp product(s) became as prominent as the other two products (Fig. 6B, T+2G, closed arrowhead). In the cells transformed with the G+3T construct, an extra 1.5-kbp product was detected (Fig. 6B G+2T, open arrowhead). Sequencing analysis revealed that this product had a short deletion in the IES (data not shown). These results indicate that the substitutions at position +2 or +3 of the left boundary change frequencies of occurrence of alternative boundaries in vivo.

Next, the 1.3-kbp products from cells transformed with the different constructs (*1, *2 and *3 in Fig. 6B) were extracted from gel and cloned, and DNA sequences of 20 clones each were analyzed. All of the sequenced 1.3-kbp products from the cells transformed with the WT construct had the same elimination boundary (Fig. 6C, WT), which exactly corresponded with the reported boundary of endogenous R-IES [39] (Fig. 6C, red arrows). In contrast, in all of the sequenced 1.3-kbp products from the cells transformed with the T+2G and G+3T constructs, the left elimination boundaries shifted one to several bases downstream (Fig. 6C, T+2G, G+3T). Interestingly, in the T+2G and G+3T constructs, the choice of the right boundary, which had no base substitutions, was also affected (Fig. 6C, T+2G, G+3T). These results may suggest that there is crosstalk between the ends of an IES during DNA elimination. This crosstalk could be established before DNA cleavage, as would be expected if Tpb2p acts as a typical DNA transposase [40], or during the repair of the excision site as suggested by Saveliev and Cox [22]. Importantly, the observed shifts of the left boundary by the T+2G and G+3T substitutions in the in vivo assay (Fig. 6C, T+2G, G+3T) correlate well with, although not identical to, the patterns of shifts of the cleavage position by the corresponding base substitutions in the in vitro Tpb2p endonuclease assay (Fig. 5B). These results, together with the facts that Tpb2p has an endonuclease activity with wide substrate specificity and is necessary for DNA elimination, strongly suggest that Tpb2p is the excisase, the enzyme that cuts IES boundaries in vivo.

## Discussion

In this study, we dissected the roles of the *Tetrahymena piggyBac* transposase-like protein Tpb2p in DNA elimination. We observed that Tpb2p has two genetically separable functions: the heterochromatin body forming activity, which resides in its cysteine-rich domain, and the DNA excision activity, which requires the endonuclease domain of Tpb2p. Furthermore, our in vitro biochemical studies indicated that the cysteine-rich domain of Tpb2p interacts with heterochromatin-specific histone modifications, and the endonuclease activity of Tpb2p has relaxed sequence specificity with its substrates. Here, we discuss how these biochemical features of Tpb2p potentially determine the reproducible occurrence of IES boundaries in vivo and how these features have been formed during the course of the evolution of DNA elimination in ciliates.

### How does Tpb2p execute the reproducible DNA elimination?

We demonstrated that the cysteine-rich domain of Tpb2p directly interacts with the N-terminal tail of histone H3, and the interaction is significantly enhanced by the heterochromatin-specific histone modifications H3K9me3 and H3K27me3 in vitro (Fig. 4). Because these histone modifications specifically occur on IESs in the developing new Mac [18], [19], Tpb2p can be recruited to IESs through its direct interaction to H3K9me3 and H3K27me3, and this recruitment may limit the occurrence of Tpb2p-endonuclease cleavage to the near surrounding heterochromatic regions. The interaction between Tpb2p and H3K9/K27me3 may specifically activate the Tpb2p-endonuclease to inhibit Tpb2p to form DNA DSB at non-IES loci. An IES is removed as one piece of DNA [22]–[24]. Therefore, although H3K9me3 and H3K27me3 likely occur throughout an IES [18], [19], the endonucleolytic cleavages of Tpb2p must be restricted to the ends of an IES. Because H3K9me3 and H3K27me3 are also bound by one of the most abundant heterochromatin components, Pdd1p [18], [19], competition of chromatin-binding sites with Pdd1p may exclude Tpb2p from the body of heterochromatin and only allow Tpb2p to bind the edges of heterochromatin regions. Alternatively, Tpb2p may localize throughout the heterochromatin segment, whereas the heterochromatin structure or some heterochromatin proteins may inhibit the action of Tpb2p endonuclease at the body of heterochromatin. Future research should clarify the spatial localization of Tpb2p on chromatin, which will help with understanding how Tpb2p acts only at the ends of IESs.

Regardless of what molecular mechanism limits the nucleolytic action of Tpb2p to the IES ends, the heterochromatin-Tpb2p interaction does not appear to be sufficient to explain the reproducible occurrence of the border of IESs because 1) histone modifications are able to determine a chromatin segment only at the level of a size of a nucleosome, whereas most of the boundaries of IESs occur within a few to several nucleotides, and 2) there are IESs in *Tetrahymena* that have sizes similar to a single nucleosome [15]. This study demonstrated that the Tpb2p endonuclease has a relaxed sequence preference for its substrate. For the longer IESs, the combination of the heterochromatin localization and the sequence-biased action likely allows Tpb2p to precisely determine the boundaries of IESs at a sub-nucleosomal level. For the shorter IESs, it is possible that the substrate preference of the Tpb2p endonuclease alone is sufficient to determine the precise boundary. Consistent with this idea, many of the shorter IESs have a common 5′-TTAA-3′ sequence at their boundaries [15] on which DNA DSB is efficiently introduced by the endonuclease Tpb2p in vitro [4].

In addition to the heterochromatin-Tpb2p interaction and sequence-biased action of the Tpb2p endonuclease, the cis-acting sequences adjacent to IESs may also play a role in reproducibly determining the border of IESs. Some IESs have cis-acting sequences located 40–50 nucleotides outside of the boundaries of IESs that are necessary in cis for the precise occurrence of DNA elimination boundaries [26], [28], although the mechanism explaining how cis-regulatory elements are involved in DNA elimination is unclear. Because Tpb2p can induce DSB at the IES boundary sequences in oligo DNAs without cis-regulatory elements in vitro (Fig. 5), cis-regulatory elements are not necessary for Tpb2p to recognize the IES boundary sequences, at least on naked DNA. The cis-acting sequences may set nucleosome positioning, and thus, Tpb2p can be recruited to a fixed chromosomal location. Alternatively, the cis-acting sequences may create a nucleosome-free region where DNA is accessible for Tpb2p.

### The relationship between the heterochromatin bodies and DNA elimination

The fact that the endonucleolytically inactive Tpb2p still supports heterochromatin body formation (Fig. 3E, “CD”) suggests that the heterochromatin bodies are not a product of DNA elimination but can be formed prior to the initiation of DNA elimination. In contrast, it has been reported that, in the absence of *TKU80*, the excision of IESs occurs without the formation of the heterochromatin bodies [10]. One fact we should consider to reconcile these seemingly incompatible observations is that the endonucleolytically inactive Tpb2p mutant was expressed in the conditional *TPB2* KO background. In the conditional *TPB2* KO locus, *TPB2* expression was under control of the *MTT1* promoter, which can be activated by addition of the cadmium ion. It is known that the *MTT1* promoter is leaky [26]. Therefore, although we used a medium containing minimum metals and we could not detect the wild-type Tpb2p by western blotting in the absence of the cadmium ion (Fig. 3B), it is still possible that undetectable amount of Tpb2p from the conditional *TPB2* KO locus induces some DNA elimination even without the cadmium ion in the medium. Nonetheless, because no circularized IES products were detected without inducing the wild-type *TPB2* expression in the conditional *TPB2* KO cells (Fig. 3H, WT rescue −Cu^2+^), leaky expression of *TPB2*, if any, causes no or very little IES excision event. Therefore, we conclude that the heterochromatin bodies can be formed without massive DNA elimination. On the other hand, the results reported by Lin et al. (2012) [10] indicate that massive DNA elimination is not sufficient to induce the formation of the heterochromatin bodies in the absence of *TKU80*. Because *TKU80* encodes a KU80 homolog, which binds and senses the end of DNA double-strand breaks, the formation of the heterochromatin bodies may be triggered by a signaling cascade down stream of the KU80-mediated DNA double-strand break sensing. A leaky expression of the wild-type Tpb2p may be enough to activate this signaling cascade, and together with the expression of the endonucleolytically inactive Tpb2p, it may induce the heterochromatin body formation.

### The domesticated *piggyBac* transposase Tpb2p and evolution of DNA elimination

DNA elimination pathways in ciliates are believed to have evolved as a genome defense mechanism against pathogenic invaders, such as transposons [41]. In *Tetrahymena*, there are several different types of transposons that do not share common boundary sequences, and the previous study demonstrated that Tpb2p is necessary for the elimination of all IESs tested, including the Tlr1 retrotransposon-like element [4]. Therefore, a single molecular mechanism including Tpb2p most likely executes the elimination of all transposons in *Tetrahymena*.

Tpb2p is evolutionarily related to *piggyBac* transposases. Tpb2p and *piggyBac* transposases share a common molecular architecture: the endonuclease domain possessing a DDD catalytic core and the zinc-finger-like cysteine-rich domain that is related to the PHD-finger motif. Although Tpb2p induces DSB at a variety of sequences (Fig. 5), *piggyBac* transposases specifically cut the 5′-TTAA-3′ sequence [34]. Because insertions of *piggyBac* transposase in vivo are negatively correlated with the presence of heterochromatin [42], the cysteine-rich domain of *piggyBac* transposase, in contrast with the domain of Tpb2p, is unlikely to interact with heterochromatin. Therefore, two important changes must have occurred in a piggyBac transposase during the evolution of DNA elimination in ciliates: loss of the strict sequence specificity for its substrate and gaining the ability to interact with the heterochromatin-specific histone H3 modifications.

Although the oligohymenophorean ciliates *Paramecium* and *Tetrahymena* use *piggyBac* transposases for DNA elimination [4], [35], the spirotrich ciliate *Oxytricha* uses Tc1/mariner class transposases for DNA elimination [43]. Therefore, the involvement of domesticated *piggyBac* transposases in DNA elimination has most likely evolved in the lineage of oligohymenophorean ciliates. Although IESs in *Tetrahymena* are not flanked by any common sequence, IESs in *Paramecium* are flanked by a 5′-TA-3′ [44], [45]. This indicates that the *Paramecium piggyBac* transposase-like protein Pgm still maintains a certain sequence specificity derived from an ancestral *piggyBac* transposase that cuts the 5′-TTAA-3′ sequence, whereas the *Tetrahymena* Tpb2p has evolved to recognize a much greater variety of sequences. Therefore, it appears that the *piggyBac* transposase has gradually lost its sequence specificity to the substrate during the evolution of oligohymenophorean ciliates.

Relaxation of the substrate specificity of Tpb2p might be compensated for by the heterochromatin-binding ability of the cysteine-rich domain of Tpb2p because heterochromatin is specifically formed on IESs prior to DNA elimination [18], [19]. Heterochromatin formation on IESs is targeted by an RNAi-related mechanism in *Tetrahymena* [18]. Because transposons in many different eukaryotes are silenced by heterochromatin formation induced by RNAi-related pathways [46], we speculate that heterochromatin formation on IESs in the *Tetrahymena* lineage evolved from an ancient transposon-silencing mechanism and co-existed in parallel with the DNA elimination, even before the involvement of a *piggyBac* transposase in the DNA elimination. Such ancient DNA elimination system might be operated by transposases encoded by eliminated transposons, like we see today in *Oxytricha* [41]. A *piggyBac* transposon in the *Tetrahymena* lineage might have first evolved to target heterochromatin, and then, its transposase might have been domesticated to overtake the roles of transposon-encoded transposases in DNA elimination.

Although DNA elimination is widely observed among most ciliates studied, the enzymes used for DNA elimination in different groups of ciliates have distinct properties and even have different transposon origins. Future studies of *Tetrahymena* Tpb2p and DNA elimination enzymes of other diverse groups of ciliates would help to further understanding of how a transposon has been domesticated for use in regulating a eukaryotic genome.

## Materials and Methods

### Strains and culture conditions

The wildtype strains B2086 and CU428 were provided by the *Tetrahymena* Stock Center (http://Tetrahymena.vet.cornell.edu/). Cells were cultured in 1×SPP medium with 2% proteose peptone at 30°C [47]. *TPB2* cKO and *TPB2* rescue strains were grown in the metal-depleted medium 1×SPPCT to minimize gene expressions from metal-inducible promoters. To make 1×SPPCT, 1×SPP medium was incubated with 5 g/100 ml of Chelex-100 resin (BioRad) with stirring for 2 hr. After filtration to remove the resin, essential trace metals were added (100×stock solution: FeCl3*H2O: 1 mg/ml, Co(NO_3_)_2_*6H_2_O, MnSO_4_*4H_2_O: 0.16 mg/ml). Mating was induced by mixing equal numbers of starved cells. Starvation was achieved in 10 mM Tris buffer (pH 7.5) for 12–16 hr at 30°C.

### Oligo DNAs

The oligo DNAs used in this study are shown in Supplementary Table S1.

### Creation of conditional *TPB2* KO (*TPB2* cKO) strains

A total of 475 bp of the *TPB2* 5′ flanking region and the first 1132 bp of the *TPB2* genomic sequence were amplified from genomic DNA with the primer pairs TN5MT 5′ fw/rv and TN5MT3′ fw/rv, respectively. The *neo5* cassette fused to an *MTT1* promoter was amplified from a pMNMM3 vector with the primers TN5MTneo fw/rv [48]. After PCR purification, the fragments were combined using overlapping PCR as described previously [49], resulting in the *TPB2* cKO construct, which was directly used for germline transformation of mating *Tetrahymena* UMPS strains at 3 hr post-mixing. The UMPS strains were created by introducing a uridine monophosphate synthase (*UMPS*) gene from *Dictyostelium* into the Mac of *Tetrahymena*. The expression of this gene makes *Tetrahymena* cells sensitive to 5-fluoroorotic acid (5-FOA). After conjugation, these strains should lose the UMPS gene because it is only in the Mac, and thus, the progeny are 5-FOA resistant. Biolistic transformation was performed as previously described [50]. After transformation of the *TPB2* cKO construct into the UMPS strains, 0.1 µg/ml cadmium chloride was added to the cells to induce TPB2 expression from the *MTT1* promoter. One paromomycin and 5-FOA resistant clone was obtained, which was confirmed as a heterozygous *TPB2* cKO strain. After mating to the WT strain, the heterozygous conditional KO strains were genotyped by PCR and crossed to each other to obtain homozygous conditional KO strains. During all matings, 0.1 µg/ml cadmium chloride was added.

### *TPB2* rescue system using *TPB2* cKO strains

A blasticidin-resistance cassette was amplified by PCR from pBla1 vector using the primers Bra1_OL_5RACErv and Bra1_OL_fw. In parallel, the *MTT2* promoter was amplified from the pDET2 vector with the primers *MTT2*_OL_fw and *MTT2*_HA_AvrII_rv. The two PCR constructs were combined with overlapping PCR as described previously [49]. The overlapping PCR product was cloned into pMNMM1 with *Avr*II and *Sal*I restriction enzymes, resulting in pMBM2M. The *TPB2* open reading frame was amplified from genomic DNA (strain B2086) using the primers *TPB2*ORF_AvrII_fw and *TPB2*ORF_MluI_rv and subsequently cloned with the enzymes *Mlu*I and *Avr*II in pMBM2M, resulting in pMBM2M-TPB2. Via site-directed mutagenesis, a catalytically dead version and cysteine-rich mutant were created from the template pMBM2M-*TPB2* using the primers *TPB2*_D297L_fw/rv, *TPB2*_D379L_fw/rv and *TPB2*_D495L_fw/rv or *TPB2*_C618A_fw/rv or C629A_fw/rv, respectively. These rescue vectors were then transformed into the somatic nucleus of two different mating types of conditional *TPB2* KO strains by ballistic transformation as described previously [50]. The transformants were selected with blasticidin and phenotypically assorted (up to 10 mg/ml). To assess the phenotype of the *TPB2* rescue strains, they were crossed with each other, and the expression of the respective rescue construct was induced by the addition of copper sulfate in a two-step procedure. Equal amounts of CuSO_4_ were added at 7 and 8 hr post-mixing, for a final concentration of 100 µM.

### Transformation of *Tetrahymena thermophila* with ribosomal vector

The Mic genomic region containing the R-IES was amplified as two overlapping pieces from the *Tetrahymena* total genomic DNA using the primers R-leftFW/R-midRV and R-midFW/R-rightRV. The two pieces were combined by overlapping PCR and cloned into the ribosomal vector pD5H8 [51] using the *Not*I restriction site. pD5H8 containing the R-IES, including the flanking regions, was electroporated into mating wild-type strains. Electroporation was performed as described previously [52] with slight modifications. Mating WT cells in 10 mM Tris (concentration: 7×10∧5 cells/ml) were used for transformation at 8.5 hr post-mixing. The cells were washed in 10 mM HEPES pH 7.5 and resuspended in 120 µl of 10 mM HEPES and then mixed with 120 µl of plasmid DNA in 10 mM HEPES (300 ng/µl) and electroporated (220 V, 50 Ω, 50 µF, exponential pulse) using a BioRad Gene Pulser MXcell. After transformation, the cells were incubated in 1×SPP medium at 30°C overnight without shaking. Transformants were selected in 100 µg/ml paromomycin.

### Immunofluorescence analysis

Cells were fixed in 3.7% formaldehyde and 0.5% Triton-X 100 for 30 min at room temperature. The cells were resuspended in 3.7% formaldehyde and 3.4% sucrose and dried on Superfrost Ultra Plus slides (Thermo Fisher). The samples were blocked for 2 hr with 3% BSA, 10% normal goat serum (Invitrogen) and 0.1% Tween 20 in PBS, followed by overnight incubation at 4°C in blocking solution containing a 1∶1000 dilution of anti-HA (Covance), 1∶2000 dilution of rabbit anti-Pdd1p (Abcam), 1∶2000 dilution of guinea pig anti-Pdd1p or 1∶2000 dilution of anti-Tpb2p antiserum. The guinea pig anti-Pdd1p antibody was obtained by immunizing a guinea pig with a peptide (CTAHRSGSRLSQIQSNANQV). The anti-Tpb2p antibody was obtained by immunizing a rabbit with N-terminal half (1 aa to 556 aa) of Tpb2p. After washing, the samples were incubated with a 1∶2000 dilution of secondary antibody against mouse or rabbit conjugated to Alexa 488 or Alexa 568 (Invitrogen). The samples were washed, incubated with 10 ng/ml of DAPI (Sigma) in PBST, and observed by fluorescent microscopy.

### DNA elimination assays

The DNA elimination assay using FISH was performed as previously described [53]. The plasmids Tlr1IntB, Tlr1 2 and Tlr1 4C1 [30] were mixed as templates to make probes against the Tlr1 IES. The labeling of the DNA with Cy3 was achieved by nick translation. Cells were fixed at 36 hr post-mixing as described above for immunofluorescence analysis.

The excision of the mse2.9- and the R-IES elements were examined by detecting circularized IESs by nested PCR using the primers listed in Supplementary Table S1.

### Progeny viability assay

Cells were mated at the cell density of 5×10^5^ cells/ml and single pairs were picked into a drop of metal depleted SPP medium (1×SPPCT). Pairs from the WT mating were picked at 7 h post-mixing. Tpb2 expression from the MTT2 cassette was induced in the rescued strains as described earlier and pairs were picked at around 10 h post mixing. As a control, WT rescue strain without addition of copper was used. At 24 h post-mixing 1×SPP medium was added to the drops to recover normal growth speed of the surviving cells. Sexual progeny formation was confirmed either by their 6-methylpurine resistance in the wild-type cells or by their blasticidin sensitivity in the rescue strains.

### Production of recombinant proteins

A codon-optimized *TPB2* coding region was amplified by PCR from the previously created vectors pGEX-*TPB2* or pGEX-*TPB2*-CD [4] using the primers T2CPfw/T2ECrv. The PCR products were cut with *Eco*RI and *Xho*I and cloned into the *Eco*RI and *Sal*I sites of the pMalC2X vector to obtain pMAL-*TPB2* and pMAL-*TPB2*-CD. To generate pMAL-*TPB2*-CRM, C618 to A and C629 to A mutations were introduced into pMAL-*TPB2* by site-directed mutagenesis using the DNA oligos T2EC C618A fw/rv and T2EC C629A fw/rv. To create the cysteine rich domain or its point mutant fused to the MBP protein, the primers *TPB2*_CRD_fw/rv were used with pMAL-*TPB2* or pMAL-*TPB2*-CRM.

The plasmids were introduced into the *E. coli* strain BL21(DE3), which was cultivated to an A_600_ of ∼0.8 and then incubated with 0.5 mM IPTG for 10 hr at 16°C. The cells were lysed in 500 mM NaCl, 80 mM Tris, pH 8.0, 0.2 mM PMSF and 1×complete proteinase inhibitor cocktail (Roche). The lysate was incubated with Amylose resin (NEB) at 4°C, washed with 500 mM NaCl and 80 mM Tris pH 8.0 and finally eluted with 500 mM NaCl, 80 mM Tris, pH 8.0, and 20 mM maltose, followed by dialysis in 20 mM Tris-HCl, pH 7.5, 100 mM KCl, 4 mM MgCl_2_, 4 mM MnSO_4_ and 10% glycerol.

### Tpb2p endonuclease assay

The endonuclease assay was performed as previously described [4]. The oligomeric DNA substrates used for the experiments are listed in Supplementary Table S1.

### Histone-peptide pull-down assay

Peptides corresponding to the N-terminal tail of histone H3 were synthesized and biotin-tagged at their C terminal with a PEG linker (Supplementary Table S2). In addition, 10 µl (bed volume) of streptavidin-coupled Dynabeads (Invitrogen) were blocked with 2% BSA in interaction buffer (10 mM Tris pH 7.5, 0.1 mM ZnSO_4_, 0.05% NP40 and 250 mM NaCl) for 1 hr at RT and then incubated with 1 µg of peptide in the blocking solution for 30 min at RT. After washing with interaction buffer and blocking again with 2% BSA 1 µg of MBP-Tpb2p-CRD was added to the beads and incubated overnight at 4°C. After five washing steps with interaction buffer, followed by two washing steps in PBS-T, the beads were resuspended in SDS-PAGE loading buffer, transferred to a fresh tube and boiled for 5 min. The samples were then separated on a 10% SDS-polyacrylamide gel, followed by a western blot. Detection was accomplished using anti-MBP antibody (NEB). The secondary antibody was coupled with an infrared dye, which was visualized with an Odyssey scanner (LI-COR Biosciences).

## Supporting Information

Figure S1 Alignment of PHD finger-like domains. The PHD finger-like cysteine-rich domains of domesticated *piggyBac* transposases in ciliates (top), *piggyBac* transposases of metazoans (middle) and PHD finger domains interacting with histone H3 (bottom) were aligned. The potential zinc-binding residues are labeled red. The two cysteine to alanine mutations (C618A, C629A) that produce the “cysteine-rich mutants” shown in Fig. 3 and Fig. 4 are indicated on top. The alignment indicates that although canonical *piggyBac* transposases and histone H3-interacting PHD fingers have eight potential zinc-binding residues that form two intermingled zinc fingers (Zn1 and Zn2), the ciliate domesticated *piggyBac* transposases have lost one potential zinc-binding residue.
(EPS)

Table S1 Oligo DNA sequences used in this study.
(TIF)

Table S2 Peptide sequences used in this study.
(TIF)

## References

[1] Orgel LE, Crick FH (1980) Selfish DNA: the ultimate parasite. Nature 284: 604–607. 7366731 10.1038/284604a0

[2] Almeida R, Allshire RC (2005) RNA silencing and genome regulation. Trends Cell Biol 15: 251–258 doi:10.1016/j.tcb.2005.03.006 15866029 10.1016/j.tcb.2005.03.006

[3] Volff J-N (2006) Turning junk into gold: domestication of transposable elements and the creation of new genes in eukaryotes. Bioessays 28: 913–922 doi:10.1002/bies.20452 16937363 10.1002/bies.20452

[4] Cheng C-Y, Vogt A, Mochizuki K, Yao M-C (2010) A domesticated piggyBac transposase plays key roles in heterochromatin dynamics and DNA cleavage during programmed DNA deletion in Tetrahymena thermophila. Mol Biol Cell 21: 1753–1762 doi:10.1091/mbc.E09-12-1079 20357003 10.1091/mbc.E09-12-1079 PMC2869380

[5] Prescott DM (1994) The DNA of ciliated protozoa. Microbiol Rev 58: 233–267. 8078435 10.1128/mr.58.2.233-267.1994 PMC372963

[6] Eisen JA, Coyne RS, Wu M, Wu D, Thiagarajan M, et al (2006) Macronuclear genome sequence of the ciliate Tetrahymena thermophila, a model eukaryote. PLoS Biol 4: e286 doi:10.1371/journal.pbio.0040286 16933976 10.1371/journal.pbio.0040286 PMC1557398

[7] Hamilton E, Bruns P, Lin C, Merriam V, Orias E, et al (2005) Genome-wide characterization of tetrahymena thermophila chromosome breakage sites. I. Cloning and identification of functional sites. Genetics 170: 1611–1621 doi:10.1534/genetics.104.031401 15956677 10.1534/genetics.104.031401 PMC1449750

[8] Fan Q, Yao M (1996) New telomere formation coupled with site-specific chromosome breakage in Tetrahymena thermophila. Mol Cell Biol 16: 1267–1274. 8622671 10.1128/mcb.16.3.1267 PMC231109

[9] Yao MC, Choi J, Yokoyama S, Austerberry CF, Yao CH (1984) DNA elimination in Tetrahymena: a developmental process involving extensive breakage and rejoining of DNA at defined sites. Cell 36: 433–440. 6319023 10.1016/0092-8674(84)90236-8

[10] Lin I-T, Chao J-L, Yao M-C (2012) An essential role for the DNA breakage-repair protein Ku80 in programmed DNA rearrangements in Tetrahymena thermophila. Mol Biol Cell 23: 2213–2225 doi:10.1091/mbc.E11-11-0952 22513090 10.1091/mbc.E11-11-0952 PMC3364183

[11] Kapusta A, Matsuda A, Marmignon A, Ku M, Silve A, et al (2011) Highly precise and developmentally programmed genome assembly in Paramecium requires ligase IV-dependent end joining. PLoS Genet 7: e1002049 doi:10.1371/journal.pgen.1002049 21533177 10.1371/journal.pgen.1002049 PMC3077386

[12] Schoeberl UE, Kurth HM, Noto T, Mochizuki K (2012) Biased transcription and selective degradation of small RNAs shape the pattern of DNA elimination in Tetrahymena. Genes Dev 26: 1729–1742 doi:10.1101/gad.196493.112 22855833 10.1101/gad.196493.112 PMC3418590

[13] Coyne RS, Stover NA, Miao W (2012) Whole genome studies of Tetrahymena. Methods Cell Biol 109: 53–81 doi:10.1016/B978-0-12-385967-9.00004-9 22444143 10.1016/B978-0-12-385967-9.00004-9

[14] Chalker DL, Yao M-C (2011) DNA elimination in ciliates: transposon domestication and genome surveillance. Annu Rev Genet 45: 227–246 doi:10.1146/annurev-genet-110410-132432 21910632 10.1146/annurev-genet-110410-132432

[15] Fass JN, Joshi NA, Couvillion MT, Bowen J, Gorovsky MA, et al (2011) Genome-Scale Analysis of Programmed DNA Elimination Sites in Tetrahymena thermophila. G3 (Bethesda) 1: 515–522 doi:10.1534/g3.111.000927 22384362 10.1534/g3.111.000927 PMC3276166

[16] Schoeberl UE, Mochizuki K (2011) Keeping the soma free of transposons: programmed DNA elimination in ciliates. J Biol Chem 286: 37045–37052 doi:10.1074/jbc.R111.276964 21914793 10.1074/jbc.R111.276964 PMC3199450

[17] Kataoka K, Mochizuki K (2011) Programmed DNA elimination in Tetrahymena: a small RNA-mediated genome surveillance mechanism. Adv Exp Med Biol 722: 156–173 doi:_10.1007/978-1-4614-0332-6_10 21915788 10.1007/978-1-4614-0332-6_10 PMC3766321

[18] Liu Y, Mochizuki K, Gorovsky MA (2004) Histone H3 lysine 9 methylation is required for DNA elimination in developing macronuclei in Tetrahymena. Proc Natl Acad Sci USA 101: 1679–1684 doi:10.1073/pnas.0305421101 14755052 10.1073/pnas.0305421101 PMC341817

[19] Liu Y, Taverna SD, Muratore TL, Shabanowitz J, Hunt DF, et al (2007) RNAi-dependent H3K27 methylation is required for heterochromatin formation and DNA elimination in Tetrahymena. Genes Dev 21: 1530–1545 doi:10.1101/gad.1544207 17575054 10.1101/gad.1544207 PMC1891430

[20] Madireddi MT, Coyne RS, Smothers JF, Mickey KM, Yao MC, et al (1996) Pdd1p, a novel chromodomain-containing protein, links heterochromatin assembly and DNA elimination in Tetrahymena. Cell 87: 75–84. 8858150 10.1016/s0092-8674(00)81324-0

[21] Smothers JF, Madireddi MT, Warner FD, Allis CD (1997) Programmed DNA degradation and nucleolar biogenesis occur in distinct organelles during macronuclear development in Tetrahymena. J Eukaryot Microbiol 44: 79–88. 9109258 10.1111/j.1550-7408.1997.tb05942.x

[22] Saveliev SV, Cox MM (2001) Product analysis illuminates the final steps of IES deletion in Tetrahymena thermophila. EMBO J 20: 3251–3261 doi:10.1093/emboj/20.12.3251 11406601 10.1093/emboj/20.12.3251 PMC150193

[23] Saveliev SV, Cox MM (1994) The fate of deleted DNA produced during programmed genomic deletion events in Tetrahymena thermophila. Nucleic Acids Res 22: 5695–5701. 7838724 10.1093/nar/22.25.5695 PMC310135

[24] Yao MC, Yao CH (1994) Detection of circular excised DNA deletion elements in Tetrahymena thermophila during development. Nucleic Acids Res 22: 5702–5708. 7838725 10.1093/nar/22.25.5702 PMC310136

[25] Taverna SD, Coyne RS, Allis CD (2002) Methylation of histone h3 at lysine 9 targets programmed DNA elimination in tetrahymena. Cell 110: 701–711. 12297044 10.1016/s0092-8674(02)00941-8

[26] Chalker DL, La Terza A, Wilson A, Kroenke CD, Yao MC (1999) Flanking regulatory sequences of the Tetrahymena R deletion element determine the boundaries of DNA rearrangement. Mol Cell Biol 19: 5631–5641. 10409752 10.1128/mcb.19.8.5631 PMC84415

[27] Godiska R, James C, Yao MC (1993) A distant 10-bp sequence specifies the boundaries of a programmed DNA deletion in Tetrahymena. Genes Dev 7: 2357–2365. 8253382 10.1101/gad.7.12a.2357

[28] Godiska R, Yao MC (1990) A programmed site-specific DNA rearrangement in Tetrahymena thermophila requires flanking polypurine tracts. Cell 61: 1237–1246. 2364428 10.1016/0092-8674(90)90688-b

[29] Shang Y, Song X, Bowen J, Corstanje R, Gao Y, et al (2002) A robust inducible-repressible promoter greatly facilitates gene knockouts, conditional expression, and overexpression of homologous and heterologous genes in Tetrahymena thermophila. Proc Natl Acad Sci USA 99: 3734–3739 doi:10.1073/pnas.052016199 11891286 10.1073/pnas.052016199 PMC122593

[30] Wells JM, Ellingson JL, Catt DM, Berger PJ, Karrer KM (1994) A small family of elements with long inverted repeats is located near sites of developmentally regulated DNA rearrangement in Tetrahymena thermophila. Mol Cell Biol 14: 5939–5949. 8065327 10.1128/mcb.14.9.5939-5949.1994 PMC359120

[31] Austerberry CF, Allis CD, Yao MC (1984) Specific DNA rearrangements in synchronously developing nuclei of Tetrahymena. Proc Natl Acad Sci USA 81: 7383–7387. 6095290 10.1073/pnas.81.23.7383 PMC392150

[32] Boldrin F, Santovito G, Formigari A, Bisharyan Y, Cassidy-Hanley D, et al (2008) MTT2, a copper-inducible metallothionein gene from Tetrahymena thermophila. Comp Biochem Physiol C Toxicol Pharmacol 147: 232–240 doi:10.1016/j.cbpc.2007.10.002 18068524 10.1016/j.cbpc.2007.10.002

[33] Keith JH, Schaeper CA, Fraser TS, Fraser MJ (2008) Mutational analysis of highly conserved aspartate residues essential to the catalytic core of the piggyBac transposase. BMC Mol Biol 9: 73 doi:10.1186/1471-2199-9-73 18694512 10.1186/1471-2199-9-73 PMC2533014

[34] Mitra R, Fain-Thornton J, Craig NL (2008) piggyBac can bypass DNA synthesis during cut and paste transposition. EMBO J 27: 1097–1109 doi:10.1038/emboj.2008.41 18354502 10.1038/emboj.2008.41 PMC2323262

[35] Baudry C, Malinsky S, Restituito M, Kapusta A, Rosa S, et al (2009) PiggyMac, a domesticated piggyBac transposase involved in programmed genome rearrangements in the ciliate Paramecium tetraurelia. Genes Dev 23: 2478–2483 doi:10.1101/gad.547309 19884254 10.1101/gad.547309 PMC2779751

[36] Bienz M (2006) The PHD finger, a nuclear protein-interaction domain. Trends Biochem Sci 31: 35–40 doi:10.1016/j.tibs.2005.11.001 16297627 10.1016/j.tibs.2005.11.001

[37] Katoh M, Hirono M, Takemasa T, Kimura M, Watanabe Y (1993) A micronucleus-specific sequence exists in the 5′-upstream region of calmodulin gene in Tetrahymena thermophila. Nucleic Acids Res 21: 2409–2414. 8506136 10.1093/nar/21.10.2409 PMC309540

[38] Austerberry CF, Snyder RO, Yao MC (1989) Sequence microheterogeneity is generated at junctions of programmed DNA deletions in Tetrahymena thermophila. Nucleic Acids Res 17: 7263–7272. 2798093 10.1093/nar/17.18.7263 PMC334806

[39] Saveliev SV, Cox MM (1996) Developmentally programmed DNA deletion in Tetrahymena thermophila by a transposition-like reaction pathway. EMBO J 15: 2858–2869. 8654384 PMC450224

[40] Dyda F, Chandler M, Hickman AB (2012) The emerging diversity of transpososome architectures. Q Rev Biophys 45: 493–521 doi:10.1017/S0033583512000145 23217365 10.1017/S0033583512000145 PMC7292550

[41] Klobutcher LA, Herrick G (1997) Developmental genome reorganization in ciliated protozoa: the transposon link. Prog Nucleic Acid Res Mol Biol 56: 1–62. 9187050 10.1016/s0079-6603(08)61001-6

[42] Wang H, Mayhew D, Chen X, Johnston M, Mitra RD (2012) “Calling cards” for DNA-binding proteins in mammalian cells. Genetics 190: 941–949 doi:10.1534/genetics.111.137315 22214611 10.1534/genetics.111.137315 PMC3296256

[43] Nowacki M, Higgins BP, Maquilan GM, Swart EC, Doak TG, et al (2009) A functional role for transposases in a large eukaryotic genome. Science 324: 935–938 doi:10.1126/science.1170023 19372392 10.1126/science.1170023 PMC3491810

[44] Steele CJ, Barkocy-Gallagher GA, Preer LB, Preer JR (1994) Developmentally excised sequences in micronuclear DNA of Paramecium. Proc Natl Acad Sci USA 91: 2255–2259. 8134383 10.1073/pnas.91.6.2255 PMC43349

[45] Arnaiz O, Mathy N, Baudry C, Malinsky S, Aury J-M, et al (2012) The Paramecium germline genome provides a niche for intragenic parasitic DNA: evolutionary dynamics of internal eliminated sequences. PLoS Genet 8: e1002984 doi:10.1371/journal.pgen.1002984 23071448 10.1371/journal.pgen.1002984 PMC3464196

[46] Grewal SI (2010) RNAi-dependent formation of heterochromatin and its diverse functions. Curr Opin Genet Dev 20: 134–141 doi:10.1016/j.gde.2010.02.003 20207534 10.1016/j.gde.2010.02.003 PMC3005588

[47] Gorovsky MA, Yao MC, Keevert JB, Pleger GL (1975) Isolation of micro- and macronuclei of Tetrahymena pyriformis. Methods Cell Biol 9: 311–327. 805898 10.1016/s0091-679x(08)60080-1

[48] Busch CJ-L, Vogt A, Mochizuki K (2010) Establishment of a Cre/loxP recombination system for N-terminal epitope tagging of genes in Tetrahymena. BMC Microbiol 10: 191 doi:10.1186/1471-2180-10-191 20626890 10.1186/1471-2180-10-191 PMC2912859

[49] Kataoka K, Schoeberl UE, Mochizuki K (2010) Modules for C-terminal epitope tagging of Tetrahymena genes. J Microbiol Methods 82: 342–346 doi:10.1016/j.mimet.2010.07.009 20624430 10.1016/j.mimet.2010.07.009 PMC2935961

[50] Bruns PJ, Cassidy-Hanley D (2000) Biolistic transformation of macro- and micronuclei. Methods Cell Biol 62: 501–512. 10503214 10.1016/s0091-679x(08)61553-8

[51] Yao MC, Yao CH (1989) Accurate processing and amplification of cloned germ line copies of ribosomal DNA injected into developing nuclei of Tetrahymena thermophila. Mol Cell Biol 9: 1092–1099. 2725489 10.1128/mcb.9.3.1092-1099.1989 PMC362699

[52] Sweet MT, Allis CD (2006) Transformation of Tetrahymena thermophila by Electroporation. CSH Protoc 2006. doi:10.1101/pdb.prot4502 10.1101/pdb.prot4502 22485896

[53] Loidl J, Scherthan H (2004) Organization and pairing of meiotic chromosomes in the ciliate Tetrahymena thermophila. J Cell Sci 117: 5791–5801 doi:10.1242/jcs.01504 15522890 10.1242/jcs.01504

