# Supplementary figures and images for: A Domesticated PiggyBac Transposase Interacts with Heterochromatin and Catalyzes Reproducible DNA Elimination in Tetrahymena
Source: PLoS Genet. 2013 Dec 12;9(12):e1004032. doi: 10.1371/journal.pgen.1004032 (PMC3861120; doi:10.1371/journal.pgen.1004032)

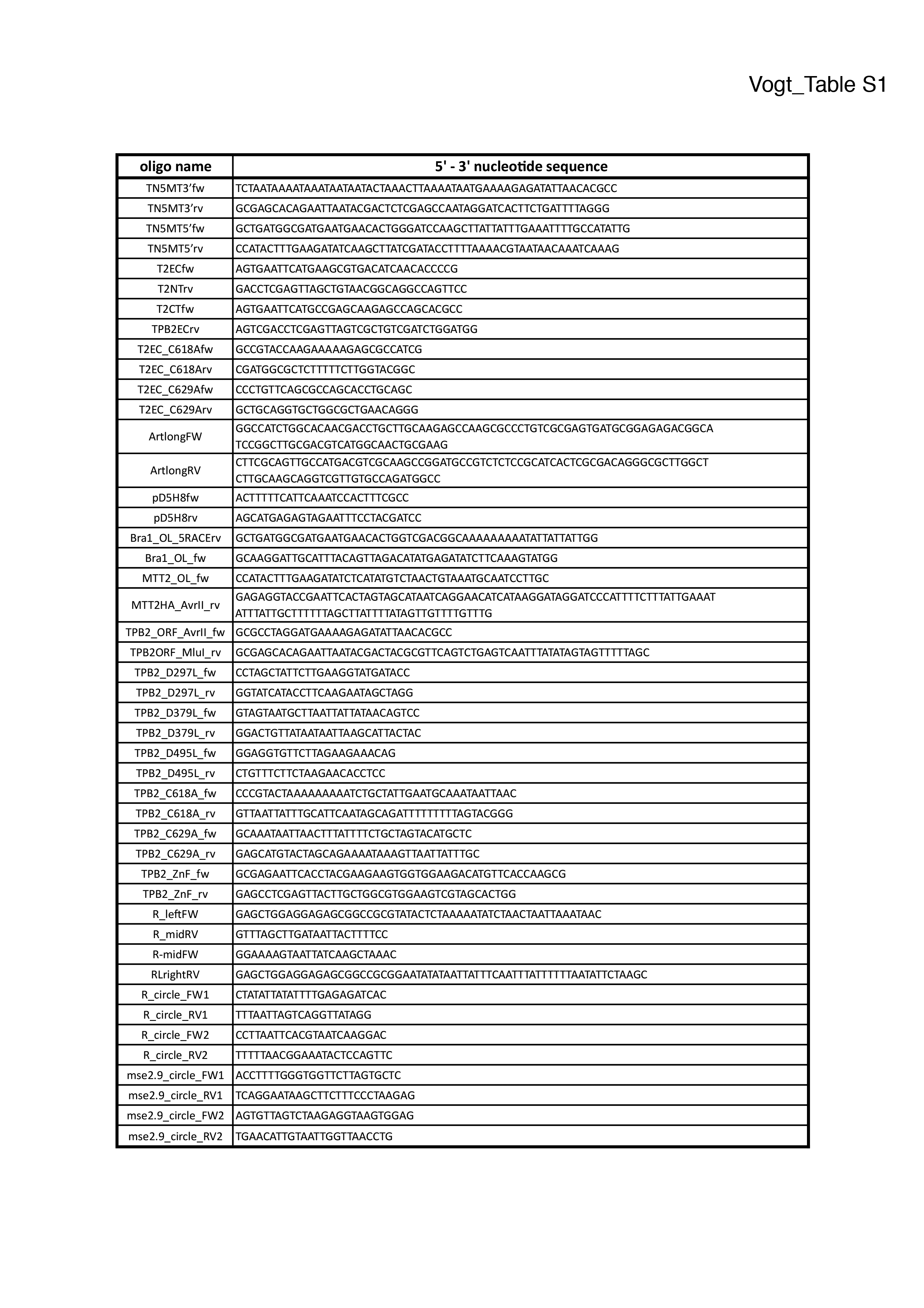

Supplement: Table S1 — Oligo DNA sequences used in this study. (TIF) [file pgen.1004032.s002.tif]

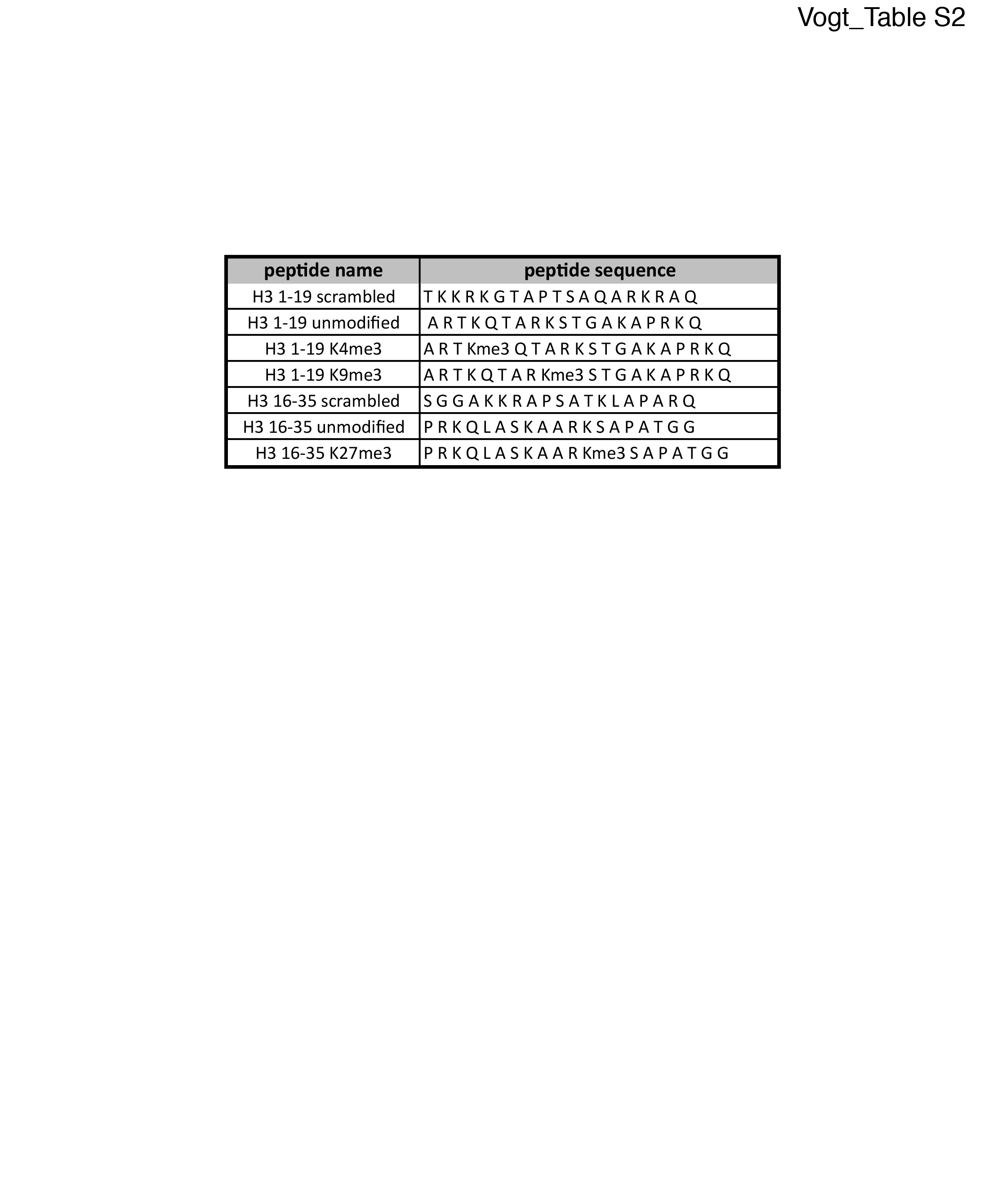

Supplement: Table S2 — Peptide sequences used in this study. (TIF) [file pgen.1004032.s003.tif]
